# Detecting expression of 5T4 in CTCs and tumor samples from NSCLC patients

**DOI:** 10.1371/journal.pone.0179561

**Published:** 2017-07-20

**Authors:** Steven R. Pirie-Shepherd, Cory Painter, Pamela Whalen, Pamela Vizcarra, Marc Roy, Jesse Qian, Tania Franks, Tim Coskran, Jon Golas, Shibing Deng, Wenyan Zhong, Eric Tucker, Dena Marrinucci, Hans-Peter Gerber, Eric L. Powell

**Affiliations:** 1 Pfizer Inc, WRD, EORCD, La Jolla, California, United States of America; 2 Pfizer Inc, WRD, IPL, Groton, Connecticut, United States of America; 3 Pfizer Inc, WRD, OTTD, Pearl River, New York, United States of America; 4 Epic Sciences, San Diego, California, United States of America; 5 Truvian Sciences, San Diego, California, United States of America; The Ohio State University, UNITED STATES

## Abstract

The fetal oncogene 5T4 is a cell surface protein, with overexpression observed in a variety of cancers as compared to normal adult tissue. The ability to select patients with tumors that express high levels of 5T4 may enrich a clinical trial cohort with patients most likely to respond to 5T4 targeted therapy. To that end, we developed assays to measure 5T4 in both tumors and in circulating tumor cells (CTCs). We identified the presence of 5T4 in both adenocarcinoma and squamous cell carcinoma of lung, in all clinical stages and grades of disease. CTCs were identified in peripheral blood from the majority of patients with NSCLC, and 5T4 was detectable in most samples. Although 5T4 was present in both CTCs and tumors in most patients, there was no concordance between relative amount in either sample type. Clinical response rates of patients treated with the therapies directed against 5T4 in early stage clinical trials, as determined by these assays, may provide important insights into the biology of 5T4 in tumors and the mechanisms of action of 5T4-targeting therapy.

## Introduction

The fetal oncogene 5T4 is a cell surface protein, with overexpression observed in a variety of cancers as compared to normal adult tissue [1]. The fetal oncogene is seen in advanced disease states and has been reported to be associated with worse prognosis in NSCLC, gastric, and ovarian cancer [2,3,4]. Recent studies [2] showed 5T4 is also observed on proliferating tumor initiating cells (TICs), and appeared to be associated with undifferentiated tumors and epithelial-mesenchymal transition (EMT), as well as a more invasive phenotype.

There have been several attempts to target 5T4 in clinical trials, and recently, there have been studies utilizing an antibody drug conjugate directed against 5T4 [5,6]. In strategies employing targeted therapy, although the presence of target may not necessarily guarantee a response to treatment, the absence of the target should be an indicator of lack of response in therapy that is truly targeted. Thus selecting patients with tumors that express high levels of the target is generally believed to increase the response rate to a targeted therapy in clinical trials. To this end, we developed two assays for use in clinical trials. One, an immunohistochemistry (IHC) assay to measure 5T4 in formalin fixed paraffin embedded tumors, the other, an assay to enumerate and measure 5T4 in circulating tumor cells (CTCs).

Circulating tumor cells (CTCs) are a recent focus of research, in part due to their relatively simple and non-invasive means of collection and their potential utility as biomarkers in cancer. They may also be studied to help further understand the metastatic process [7].

It is hypothesized that epithelial cells undergo Epithelial-Mesenchymal Transition (EMT) as they lose their ability to form cell-cell interactions, gain motility [8] and potentially become CTCs. The EMT is not believed to be a binary state, and many CTCs may express a range of epithelial or mesenchymal markers [9,10]. Although the metastatic process is complex and currently poorly understood, insight into the role CTCs may play in the metastatic process should be informative in appreciating the potential clinical significance of CTCs. Invasion and metastasis are hallmarks of malignancy, and current hypotheses involve cells undergoing the epithelial mesenchymal transition and perhaps transition to a more metastatic phenotype.

In this present study, we have performed bioinformatics analysis to demonstrate 5T4 mRNA expression in both adenocarcinoma and squamous cell carcinoma of lung. Using 5T4-specific antibodies, we developed an IHC assay to detect 5T4 in NSCLC and an immunofluorescence assay to detect 5T4 in CTCs.

We used these assays to profile matched samples obtained from treatment naïve NSCLC patients. CTCs were enumerated and evaluated for 5T4 in peripheral blood, as were matched tumor resections. The IHC assay was developed using cell lines, xenografts, and snap frozen NSCLC samples that were characterized for 5T4 via quantitative reverse transcriptase polymerase chain reaction (qRT-PCR) and western blot. The impact of pre-analytical variables, such as time to fixation and time of fixation, on 5T4 membrane detection was also evaluated. We present data characterizing CTC enumeration as well as the detection and relative expression of 5T4 in CTCs and tumors from NSCLC patients and discuss the correlations between the presence of the fetal oncogene in CTCs and matched tumor resections.

## Results

### Expression of 5T4 in NSCLC tumor

**Fig 1:**
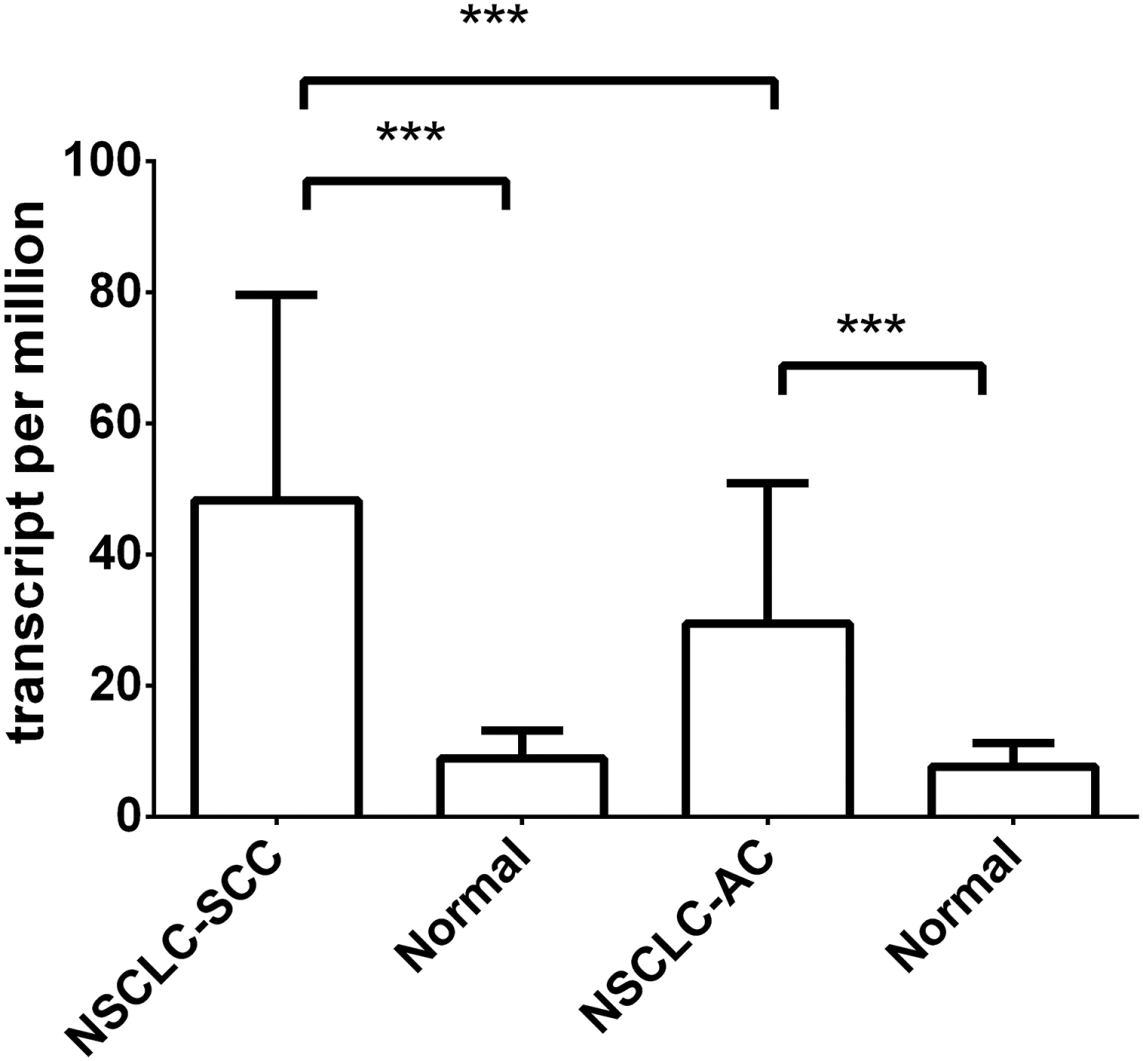
Elevated expression of 5T4 in NSCLC tumor tissue as compared to normal. 5T4 expression in NSCLC, measured in transcripts per million by RNASeq was obtained from the cancer genome atlas (TCGA) Portal. Differential expression of 5T4 expression level in Lung adenocarcinoma (n = 526) and Lung squamous cell carcinoma tumor types (n = 501) were compared to their matched normal samples (n = 58 and 51 respectively). Significance was determined using ANOVA.

Bioinformatics analysis, derived from data generated by the TCGA Research Network: (http://cancergenome.nih.gov.proxy1.athensams.net/.) with regard to NSCLC samples (n = 1037), demonstrated elevated expression of 5T4 mRNA in both adenocarcinoma and squamous cell carcinoma as compared to control tissue samples (n = 109) (Fig 1). Squamous cell carcinoma appeared to have 1.6 fold higher mean expression of 5T4 than adenocarcinoma samples. Studies were conducted to evaluate the relationship between mRNA and protein.

### Development of an Immunohistochemistry assay specific for 5T4

**Fig 2:**
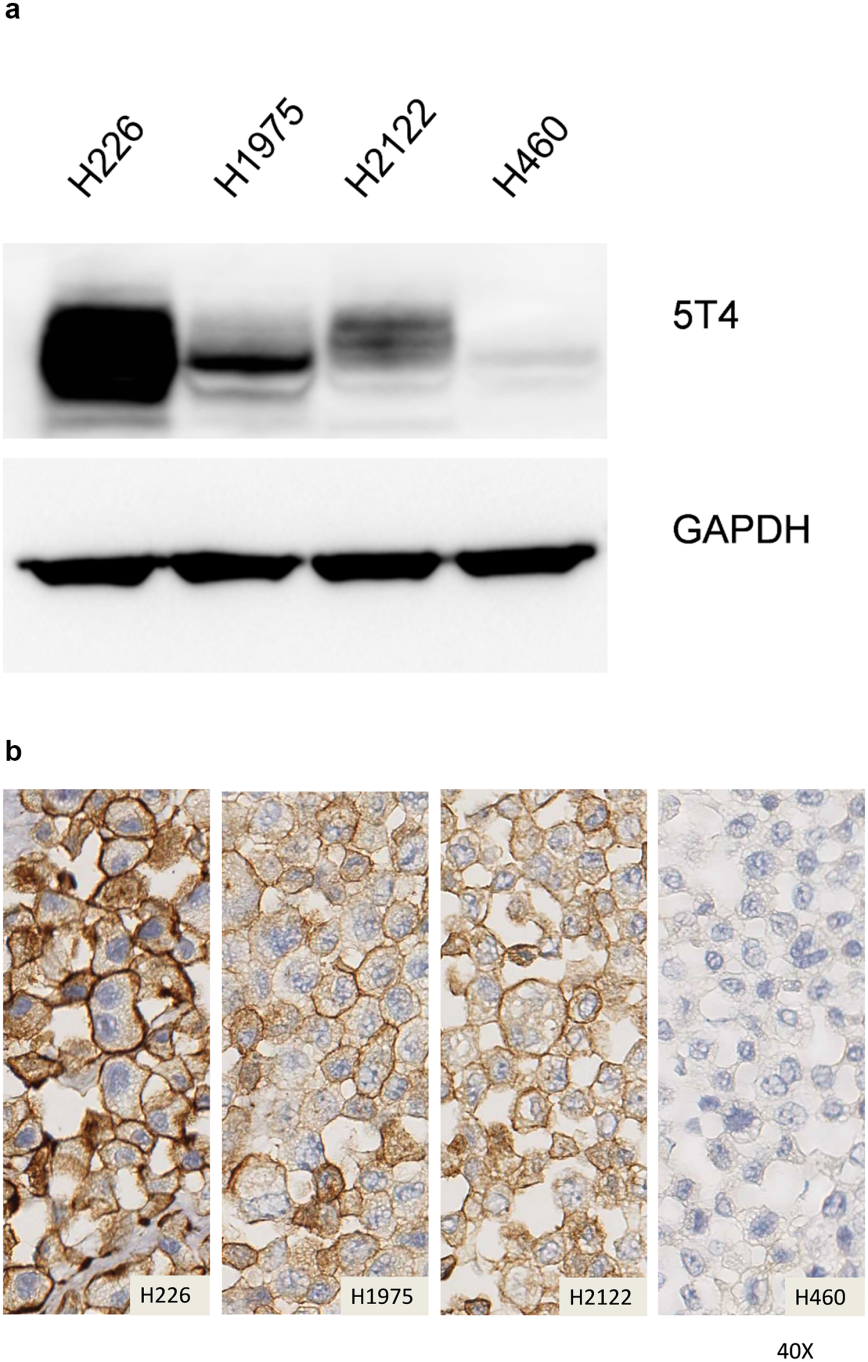
Expression of 5T4 in characterized cell lines. **A**- Western blot of the four control cell lines representing high (H226), moderate (H1975), low (H2122), and no (H460) 5T4 protein levels. The 5T4 protein is associated with a molecular weight of 72 kDa. The 5T4 band densitometry was normalized against GAPDH. Note that H226 cells have the most intensely staining 5T4 band, whereas lower levels can be seen in H1975 and H2122 cells. H460 cells appear to contain no 5T4 protein as assessed using this technique. Data was generated as described in Materials and Methods. **B**- Immunocytochemistry of the four control cell lines representing high (H226), moderate (H1975), low (H2122), and no (H460) 5T4 protein levels. Note that H226 cells have clear circumferential staining of the membrane, which is diminished in the H1975 and H2122 cells, disappearing completely in the H460 cells. Cells were prepared and stained as described in Materials and Methods.

The Cancer Cell Line encyclopedia was queried for 5T4 mRNA expression as described in Materials and Methods. Cell lines representative of relatively negative, low, moderate, and high expression (NSCLC cell lines, H460, H2122 H1975, and H226, respectively) were chosen for further characterization. The overall ranking was confirmed by qRT-PCR, western blot (Fig 2a), and immunocytochemistry analysis (Fig 2b).

The data are tabulated (Table 1), and the Spearman correlations confirming the ranking are shown in (Table 2).

**Table 1:** Expression of 5T4 in control cell lines as measured by qRT-PCR, Western Blot and Immunocytochemistry.

Cell Line	qRT-PCR	Western Blot	ICC
H226	5.11	1.00	4
H1975	1.24	0.09	3
H2122	1.09	0.10	2
H460	0.02	0.00	1

The qRT-PCR values are indexed relative to a Universal human RNA control, Western Blot data is indexed to GAPDH and relative to the values obtained for H226, the Immunocytochemistry ranking of the cell lines is based upon visual inspection of the membrane staining.

**Table 2:** Spearman Correlations of 5T4 expression as measured by Immunocytochemistry and compared to qRT-PCR and Western Blot Analysis.

Parameter	Parameter	P value	Rank R
ICC	qRT-PCR	8.3e-2	1.0
ICC	Western Blot	3.3e-1	0.8

Spearman Correlations were calculated using SpotFire 3.3.3 (Build 3.3.3.14, TIBOC, Boston, MA)

The Control cell lines were grown as xenografts, resected at ~500 mm^3^ and subjected to controlled ischemia and fixation as described in Materials and Methods. The pre-fixation xenografts were characterized for 5T4 expression by qRT-PCR and western blot.

**Fig 3:**
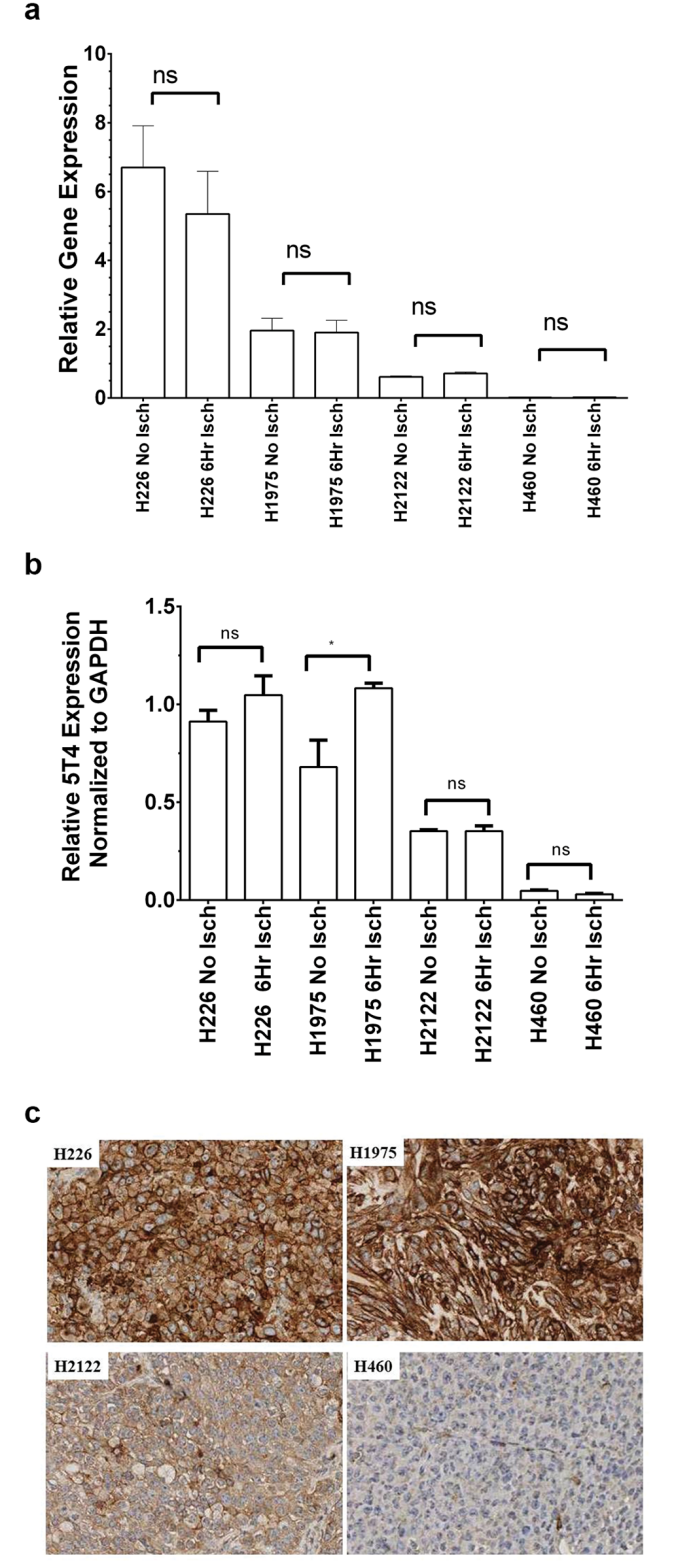
Effect of Ischemia and fixation on 5T4 expression in characterized xenografts models. **A**- The four control xenografts models representing high (H226), moderate (H1975), low (H2122), and no (H460) 5T4 protein levels, were grown in mice as described in Materials and Methods, resected and subjected to either no ischemia or 6 hours ischemia. Expression levels are indexed to GAPDH as a housekeeping gene. The expression of 5T4 is highest in the H226 cells, and is unaffected by ischemia. H1975 and H2122 cells have reduced expressions of 5T4, which is again unaffected by ischemia. H460 cells appear to have no measurable 5T4 expression as determined by this technique. The expression levels of 5T4 were assessed using qRT-PCR as described in Materials and Methods. **B**- The four control xenografts models representing high (H226), moderate (H1975), low (H2122), and no (H460) 5T4 protein levels, were grown in mice as described in Materials and Methods, resected and subjected to either no ischemia or 6 hours ischemia. The expression of 5T4 is highest in the H226 cells, and is unaffected by ischemia. H1975 cells have reduced expression of 5T4 as compared to H226 cells, and there does appear to be a slightly significant increase in protein expression under the influence of ischemia. H2122 cells have reduced expressions of 5T4, which is again unaffected by ischemia. H460 cells appear to have no measurable 5T4 expression as determined by this technique. Overall, there was no evidence of a systemic change in 5T4 expression induced by ischemia across the 4 models analyzed. The expression levels of 5T4 were assessed using Western Blot analysis as described in Materials and Methods. Protein levels are indexed to GAPDH as a housekeeping gene. **C**- The four control xenografts models representing high (H226), moderate (H1975), low (H2122), and no (H460) 5T4 protein levels, were grown in mice as described in Materials and Methods, resected and fixed. The expression levels of 5T4 were then assessed using IHC analysis as described in Materials and Methods. Staining of 5T4 in H226 xenografts is robust, showing strong circumferential staining of the cells in the model. Strong staining is also seen in the H1975 model. Reduced staining is seen in the H2122 xenograft, with fainter staining of the membranes. No overt membrane staining is seen in the H460 xenograft model.

The qRT-PCR data were plotted as histograms (Fig 3a). Ischemia had no significant effect on gene expression levels in these models. The western blot data are plotted (Fig 3b). There was no significant reduction in protein levels as a result of ischemia in these samples. An apparent statistically-significant increase in 5T4 in one cell line model (H1975) was observed. However, this appeared to be more likely due to necrosis of the non-ischemic xenograft causing a reduction in overall protein levels, including 5T4 and GAPDH, and an altered ratio of 5T4-to-GAPDH rather than a specific induction of 5T4 protein caused by ischemia.

The post-fixation xenografts were further characterized by IHC as described in Materials and Methods. Representative images of IHC showing membrane staining of each representative xenograft model is shown in Fig 3c. Membrane staining was neither eliminated nor substantially altered by ischemia or fixation at the respective time points.

The data for the negative, low, moderate, and high 5T4-expressing models are presented in Table 3.

**Table 3:** Effect of ischemia and fixation on 5T4 membrane staining as measured by Immunohistochemistry in xenograft models.

Model	Ischemia (hrs)	Fixation (hrs)	2x	4x	10x	20x
H460 (negative)	0	8	NEG	NEG	NEG	NEG
H460 (negative)	0	24	NEG	NEG	NEG	NEG
H460 (negative)	6	8	NEG	NEG	NEG	NEG
H460 (negative)	6	24	NEG	NEG	NEG	NEG
H2122 (Low expressing)	0	8	NEG	NEG	RMS/GMS	PMS
H2122 (Low expressing)	0	24	NEG	NEG	RMS/GMS	PMS
H2122 (Low expressing)	6	8	NEG	NEG	RMS/GMS	PMS
H2122 (Low expressing)	6	24	NEG	NEG	RMS/GMS	PMS
H1975 (Moderate expressing)	0	8	NEG	PMS/GMS	RMS	PMS
H1975 (Moderate expressing)	0	24	NEG	PMS/GMS	RMS	PMS
H1975 (Moderate expressing)	6	8	NEG	PMS/GMS	RMS	PMS
H1975 (Moderate expressing)	6	24	NEG	PMS/GMS	RMS	PMS
H226 (High expressing)	0	8	DMS	DMS	DMS	DMS
H226 (High expressing)	0	24	DMS	DMS	DMS	DMS
H226 (High expressing)	6	8	DMS	DMS	DMS	DMS
H226 (High expressing)	6	24	DMS	DMS	DMS	DMS

NEG = negative, RMS = rare membrane staining, GMS = geographic membrane staining, PMS = patchy membrane staining, DMS = diffuse membrane staining

### Characterization and correlation of 5T4 expression in human NSCLC tumors by western blot, qRT-PCR and IHC

Malignant epithelial tumors of lung (n = 21) were prospectively procured and prepared for western blot and TLDA analysis as described in Materials and Methods. Tumor tissue samples were processed, thawed, and fixed as described in Materials and Methods. The samples were immunolabeled for 5T4, and the stained sections H-scored by an anatomic pathologist in the usual manner.

Briefly, malignant cell membranes were evaluated for the extent of immunoreactivity using control cell lines as intensity guides and scored using the following formula: 3 x percentage of strongly staining membranes + 2 x percentage of moderately staining membranes + percentage of weakly staining membranes, yielding a range from 0 to 300. IHC H-scores were examined for correlation with qRT-PCR (TLDA) and western blot results. IHC H-score values, qRT-PCR data, and the Western blot data can be seen in Table 4.

**Table 4:** Profiling of 5T4 Expression in human NSCLC tumors by western blot, TLDA and IHC.

Sample #	Tumor Type	qRT-PCR	Western Blot	IHC H-score
1	SCC	0.50	0.01	0
2	AC	2.95	0.03	35
3	AC	3.77	0.01	0
4	AC	3.94	0.04	35
5	AC	4.00	0.02	130
6	SCC	4.41	0.04	30
7	AC	6.10	0.02	55
8	SCC	6.48	0.17	120
9	AC	6.82	0.04	107
10	AC	7.20	0.03	82
11	SCC	7.29	0.10	30
12	SCC	7.53	0.13	120
13	SCC	10.35	0.05	72
14	AC	10.91	0.01	224
15	SCC	11.80	0.04	75
16	SCC	14.51	0.04	113
17	SCC	15.32	0.09	105
18	SCC	15.53	0.10	137
19	AC	16.00	0.04	135
20	AC	22.26	0.01	175
21	AC	26.56	0.06	65

NSCLC is Non-small cell lung cancer, AC is adenocarcinoma, SCC is squamous cell carcinoma. IHC is ImmunoHistoChemistry. The qRT-PCR values are expressed as fold expression greater than seen in normal lung tissue, Western Blot values are relative to GAPDH. H-score was determined by a pathologist.

Spearman values were calculated for the correlation of the ranking of the samples as determined by IHC H-score, qRT-PCR, and Western blot analysis for the entire sample set of 21 and for squamous cell carcinoma and adenocarcinoma case subsets.

There was a positive correlation between IHC H-score and qRT-PCR in the whole sample set as well as within the individual adenocarcinoma and squamous cell carcinoma sample subsets. There was positive correlation between IHC H-score and western blot data for the whole sample set and the squamous cell carcinoma subset, but the correlation between the IHC -score and western blot data from the adenocarcinoma subset was negative. This discrepancy is discussed in the Conclusions Section. These correlations can be seen in Table 5.

**Table 5:** Spearman Correlations of H-score to TLDA and western blot data for human NSCLC tumors.

Sample	Parameter 1	Parameter 2	P value	Rank R
NSCLC (all)	H-score	Western Blot Values	0.633	0.11
NSCLC (all)	H-score	qRT-PCR Values	0.003	0.61
NSCLC (SCC)	H-score	Western Blot Values	0.012	0.66
NSCLC (SCC)	H-score	qRT-PCR Values	0.058	0.62
NSCLC (AC)	H-score	Western Blot Values	0.483	-0.19
NSCLC (AC)	H-score	qRT-PCR Values	0.0182	0.69

Spearman Correlations were calculated using SpotFire 3.3.3 (Build 3.3.3.14, TIBOC, Boston, MA)

**Fig 4:**
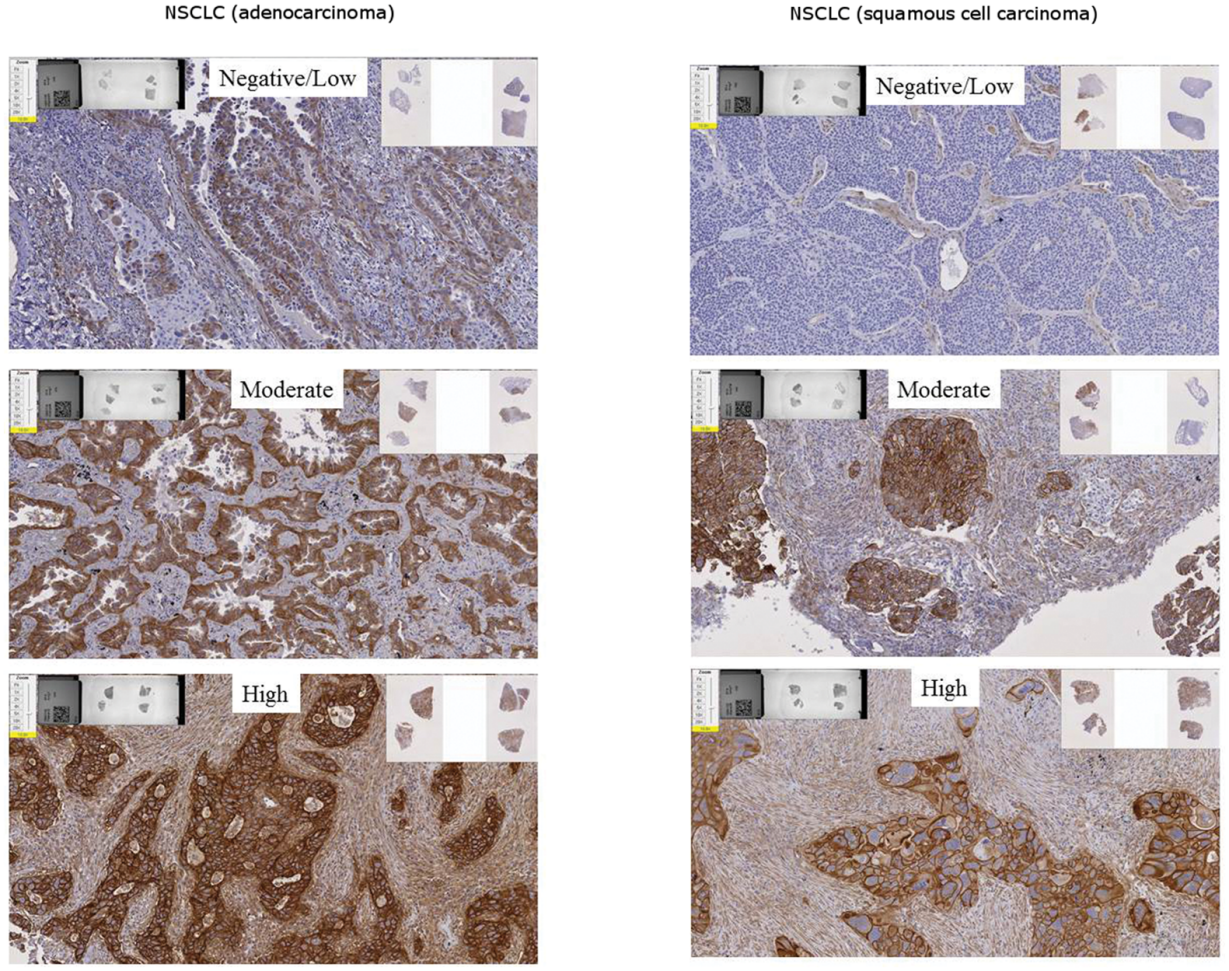
Negative/Low, Moderate and High staining of NSCLC tumor samples for 5T4. Representative images from the analytical validation sample set of human NSCLC (squamous and adenocarcinoma) tumor samples designated as Negative/Low, Moderate or High for 5T4 expression, processed and stained using the IHC assay as described in Materials and Methods. Samples were selected from a prospectively collected cohort of 24 samples that had been analyzed for 5T4 expression using qRT-PCR and Western Blot analysis, and ranked according to 5T4 expression. The samples identified as Negative/Low using orthogonal techniques have small amount of staining, whereas the samples identified as Moderate and High demonstrate increasing intensity of staining with circumferential staining of cell membranes.

Representative images of samples that demonstrate negative/low, moderate, and high membrane staining of 5T4 in NSCLC adenocarcinoma or squamous cell carcinoma tumor samples can be seen in Fig 4.

### Analysis of 5T4 expression in a small cohort of prospectively collected NSCLC samples

The positive correlation between the IHC assay, and qRT-PCR expression data, and much of the western blot data allowed us the confidence to use the IHC assay to measure 5T4 expression in a second small sample set (n = 35) of prospectively collected FFPE NSCLC tumor samples with matching blood samples drawn prior to tumor resection.

Tissue was collected, processed and stained for 5T4 expression as described in Materials and Methods. Staining was assessed by a board certified anatomic pathologist, and an IHC H-score was generated for each sample (Table 6) as described in Materials and Methods.

**Table 6:** Expression of 5T4 in the tumor and the CTC compartment of samples obtained from patients with NSCLC.

Type	IHC H-score	CTCs per mL Blood	CTC H-Score:	Cluster-Weighted H-Score:	5T4 Burden per mL Blood	Cluster Weighted 5T4 Burden per mL Blood	% 5T4 negative CTCs per mL Blood
AC	0	32	47	64	69	210	53
SCC	0	71	74	102	268	788	35
AC	2	10	38	45	15	26	63
AC	3	8	138	138	106	106	38
AC	10	3	0	0	7	7	75
SCC	14	65	29	51	82	273	74
AC	20	9	12	12	3	3	88
AC	20	0	0	0	0	0	NA
SCC	20	346	35	49	506	1018	68
AC	30	13	61	78	35	100	39
AC	31	58	97	110	269	924	9
AC	31	33	74	108	118	448	35
AC	33	34	78	98	128	353	28
AC	35	71	47	63	129	221	54
AC	35	28	15	21	17	31	85
SCC	36	7	20	17	4	4	80
AC	38	39	43	44	56	89	57
AC	45	14	24	36	12	28	76
AC	50	1	0	0	0	0	100
AC	50	247	69	99	784	2945	37
SCC	56	1	0	0	4	4	0
AC	60	0	0	0	0	0	NA
AC	60	2	0	0	89	89	0
SCC	61	16	63	64	149	167	47
AC	65	22	19	42	19	56	85
AC	70	8	86	89	31	43	14
AC	73	25	81	97	79	182	24
SCC	82	200	47	67	382	1176	54
SCC	91	2	0	0	21	21	50
AC	115	0	0	0	0	0	NA
SCC	130	94	68	82	397	615	53
AC	140	125	67	98	389	1013	42
AC	140	35	33	42	71	122	74
AC	150	45	36	53	68	172	64
SCC	169	4	0	33	7	7	50

NSCLC is Non-small cell lung cancer, AC is adenocarcinoma, SCC is squamous cell carcinoma, CTC parameters are as defined in materials and methods.

**Fig 5:**
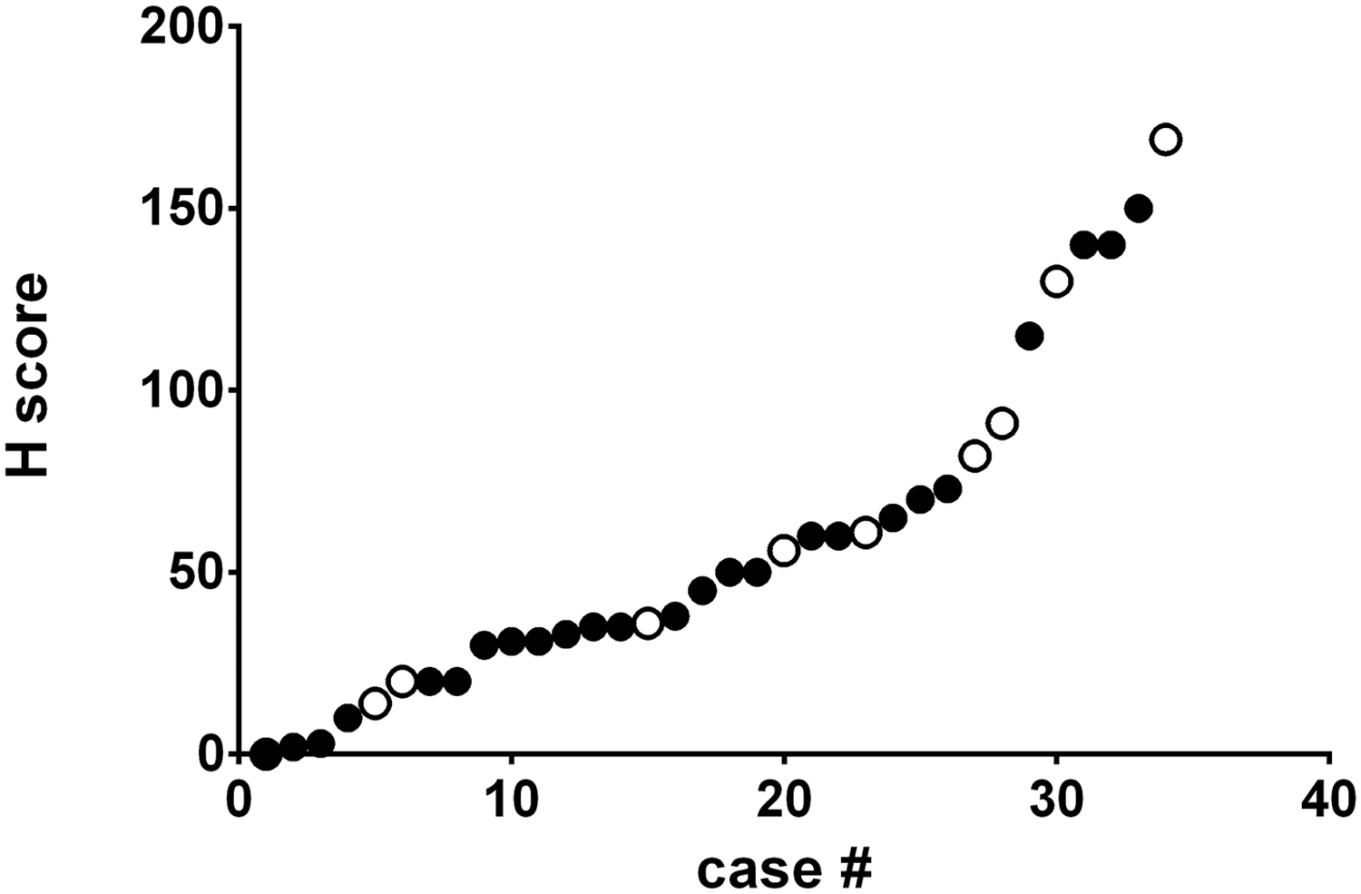
H-scores of 5T4 in adenocarcinoma and squamous cell carcinoma NSCLC samples. NSCLC tumor samples from a second cohort were prospectively collected, processed, fixed and stained as described in Materials and Methods. The samples were examined by an anatomic pathologist and each sample was given an H-score which was used to generate a rank ordered distribution. These were then plotted as a continuous distribution.

There were 25 adenocarcinoma samples and 10 squamous cell carcinoma samples in the data set. The adenocarcinoma samples had an IHC H-score of 52±9 (average ± SEM), with IHC H-score values ranging from 0 to 150 (Fig 4a). The squamous samples had an IHC H-score of 66±17 (average ± SEM) with IHC H–score values ranging from 0–169 (Fig 5). There were no significant differences between the mean IHC H-score values for each indication (p = 0.49).

Further analysis of the distribution of IHC H-score was performed by noting the clinical stage and grade of tumor within each subtype. The adenocarcinoma sample set contained specimens representing clinical stages 1A, 1B, 2A, 2B and 3A. There was no significant difference in IHC H-score values between the clinical stages in the adenocarcinoma samples (Table 7).

**Table 7:** IHC H-score in adenocarcinoma and squamous cell carcinoma by clinical stage.

subtype	1A	1B	2A	2B	3A	p value
adenocarcinoma	46± 12	72± 22	36± 14	37± 20	80± 60	0.799
squamous cell carcinoma	60± 36	56± 36	104± 33			0.671

IHC H-score values were generated by a pathologist. Statistical analysis was performed using Graphpad Prism as described in materials and methods

The squamous cell carcinoma sample set contained specimens representing clinical stage 1A, 1B and 2A. There were no significant differences associated with clinical stage in the squamous cell carcinoma samples (Table 7), although there was a non-significant increase in IHC H-score between clinical stage 1 and clinical stage 2.

The sample set could also be categorized by grade. There were no significant differences in the IHC H-score between grades in either the adenocarcinoma and squamous cell carcinoma samples (Table 8).

**Table 8:** IHC score in adenocarcinoma and squamous cell carcinoma by grade.

subtype	poorly differentiated	moderately differentiated	well differentiated	P value
adenocarcinoma	42± 9	53± 12	72± 24	0.601
squamous cell carcinoma	72± 23	74± 27		0.999

### Enumeration of 5T4-expressing CTCs

An assay to detect CD45-/CK+/DAPI+/5T4+ cells was developed as described in Materials and Methods using the same characterized control cell lines as used in the development of the IHC assay.

**Fig 6:**
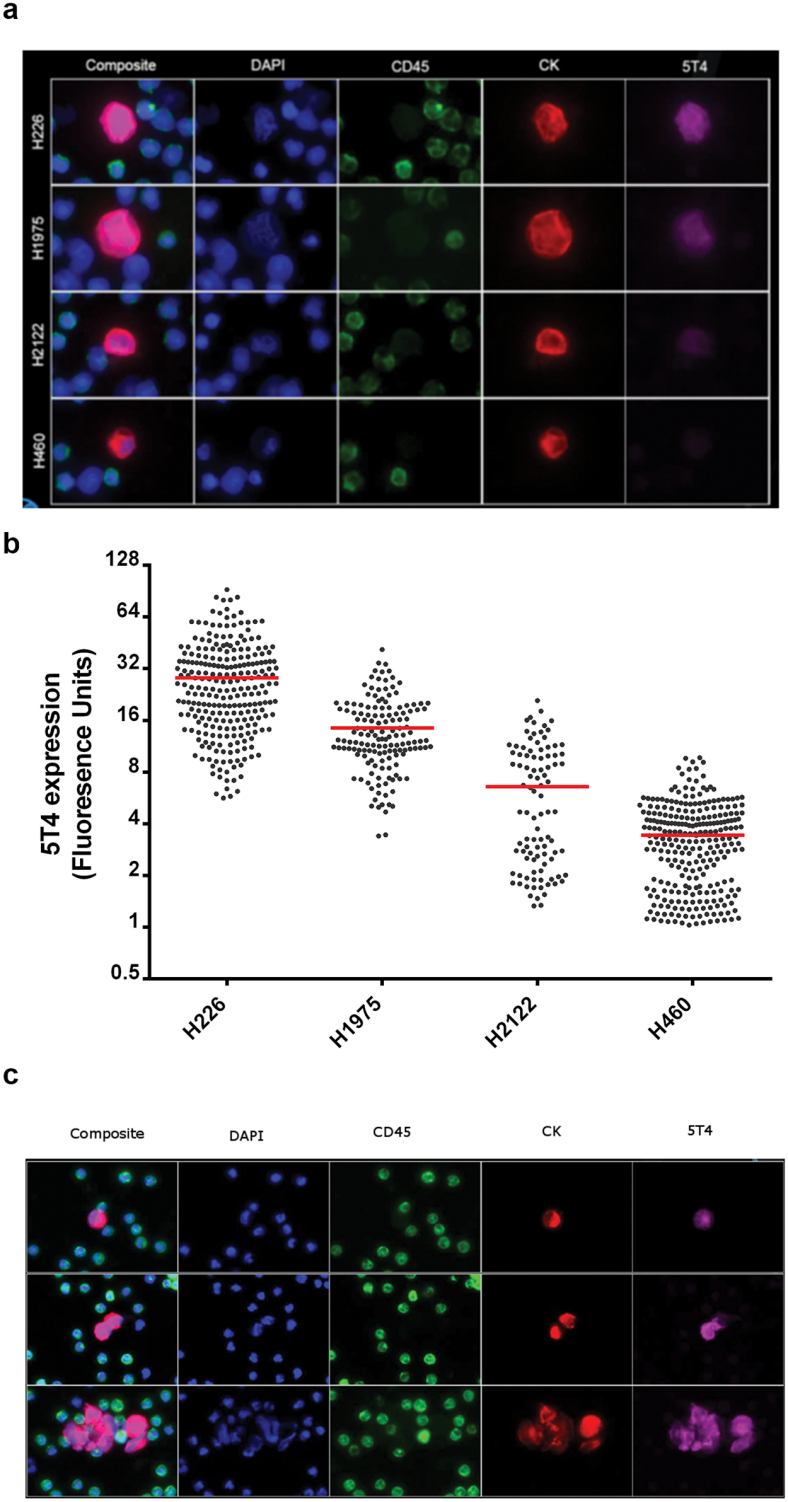
Enumeration and characterization of 5$t-expressing CTCs. **A**- Characterized control cell lines representing high (H226), moderate (H1975), low (H2122) and negative (H460) 5T4 staining were prepared and spiked into healthy human blood samples as described in materials and methods. The blood was then used to generate slides and stained with 4',6-diamidino-2-phenylindole (DAPI) to identify cell nuclei, and antibodies against CD45, cytokeratin (CK), and 5T4. The individual images were merged to form a composite image. As can be seen, the H226 cells demonstrate the most intense 5T4 staining (magenta), and this staining decreases in the H1975, and the H2122 cells. H460 cells do not appear to have 5T4 staining using this technique. All the control cells have equivalent staining using the cytokeratin (CK) specific antibodies. None of the control cells exhibit CD45 expression. **B**- Characterized control cell lines representing high (H226), moderate (H1975), low (H2122) and negative (H460) 5T4 staining were prepared and spiked into healthy human blood samples as described in materials and methods. The blood was then used to generate slides and stained with 4',6-diamidino-2-phenylindole (DAPI) to identify cell nuclei, and antibodies against CD45, cytokeratin (CK), and 5T4. The level of 5T4 in individual cells on each slide was quantified and is plotted, which each dot representing an individual cell. 5T4 is measured using fluorescence units. The red line represents the mean 5T4 expression for each cell type. The mean value obtained for H460 cells was used as a cutoff threshold to define negativity for 5T4 expression. Note that even though these control cells are clonal, there is still a range of 5T4 expression seen within each control cell line. **C**- Cells were processed and stained using the Epic Sciences platform as described in Materials and Methods. Nuclei are visualized in blue using 4',6-diamidino-2-phenylindole (DAPI), CD45 staining is represented in green, cytokeratin staining is represented in red, and 5T4 staining is represented in magenta. The 5T4 channel and the overlay (composite) indicate that 5T4 staining is seen in the membrane and cytoplasm, as is expected. Images are three separate fields from one patient. Note that this patient had CTCs that were either single, or in clusters of 2 or more cells. The presence of clusters of CTCs is seen in many patients.

Blood samples, matched to the tumor samples, were collected from the NSCLC patients, processed, stained and analyzed as described in Materials and Methods. This assay could detect either single CTCs or clusters of CTCs and was also capable of detecting (Fig 6a) and quantifying (Fig 6b) variable levels of 5T4 expression in characterized control cell lines and these values were quantifiable. Images from representative fields from one of the NSCLC samples in this study can be seen in Fig 6c.

**Fig 7:**
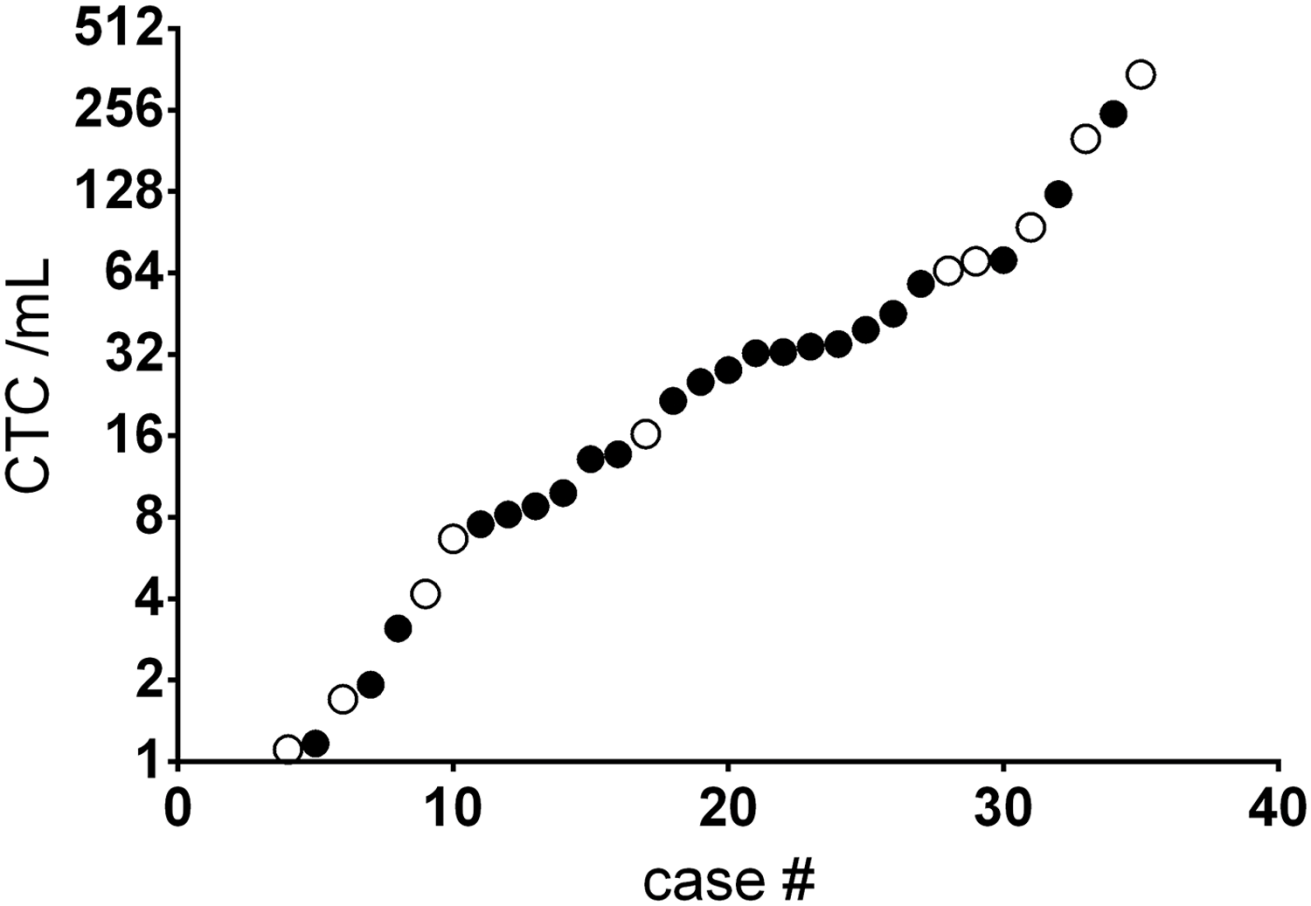
Enumeration of 5T4 expressing CTC/mL in adenocarcinoma and squamous cell carcinoma NSCLC samples. Samples were processed, stained and enumerated as described in Materials and Methods. The number of CTCs per mL ranged from 0 to over 200 per patients in this small study, demonstrating the heterogeneity in CTC numbers between patients with NSCLC. The distribution within adenocarcinoma (●) and squamous cell carcinoma (○) is noted.

The CTCs in each sample were enumerated, regardless of 5T4 expression, and ranged from 0 to 247 cells/ml in the adenocarcinoma samples, and from 1–346 cells/ml in the squamous cell carcinoma samples (Fig 7, and Table 6).

Not all the enumerated CTCs (defined as CD45-/CK+/DAPI+) in each sample expressed 5T4, about 50% of CTCs in both squamous and adenocarcinoma samples expressed 5T4 to varying degrees (Table 6). The mean number of CTCs per ml in the adenocarcinoma samples was 30±8, and 55±25 in the squamous cell carcinoma samples (Table 9).

**Table 9:** CTC metrics in adenocarcinoma and squamous cell carcinoma blood samples derived from CD45-/CK+/DAPI+/5T4+ cells.

	subtype		
metric	adenocarcinoma	squamous cell carcinoma	P value
CTC #	30±8	55±25	0.741
CTC H-score	56±7	50±7	0.705
Cluster weighted CTC H-score	70±8	65±11	0.876
5T4 Burden/mL	96±33	168±57	0.255
Cluster weighted 5T4 burden/mL	276±118	389±131	0.255

Data was generated using the Epic Sciences CTC assay as described in materials and methods. Metrics are as defined in the materials and methods. Statistics were performed by Graphpad Prism as described in materials and methods.

There was no statistically significant difference between the numbers of CTCs in adenocarcinoma and squamous cell carcinoma samples (p value = 0.741).

The number of CTCs was also calculated in the samples categorized by grade or clinical stage. These data can be seen in Tables 10 and 11. There is a non-significant reduction in the number of CTC in both adenocarcinoma and squamous cell carcinoma going from poorly differentiated tumors to well differentiated tumors (Table 10). There was a non-significant reduction in the number of CTCs in the adenocarcinoma samples as stage progressed from 1A to 1B and beyond (Table 11); such a decrease was not seen in the squamous cell carcinoma samples also (Table 11).

**Table 10:** CTC metrics by tumor grade.

subtype	metric	poorly differentiated	moderately differentiated	well differentiated	P value (Kruskal-Wallis)
adenocarcinoma	CTC #	58±38	28±8.10	22±18	0.759
	CTC H-score	58±20	32±6.70	61±31	0.288
	Cluster weighted CTC H-score	66±22	43±9.10	66±34	0.504
	5T4 Burden/mL	174±123	68±23	100±85	0.829
	Cluster weighted 5T4 burden/mL	559±478	168±62	322±301	0.927
					P value (Mann-Whitney)
squamous cell carcinoma	CTC #	160±74	17±9.8		0.257
	CTC H-score	38±14	29±12		0.609
	Cluster weighted CTC H-score	50±18	48±18		0.814
	5T4 Burden/mL	322±110	48±23		0.257
	Cluster weighted 5T4 burden/mL	703±261	113±48		0.257

**Table 11:** CTC metrics by tumor clinical stage.

		clinical stage					
subtype	metric	1A	1B	2A	2B	3A	p value (Kruskal-Wallis)
adenocarcinoma	CTC #	62±23	19±9.2	4.6±2	19±10	18±18	0.085
	CTC H-score	50±7	36±18	38±34	56±15	17±17	0.435
	Cluster weighted CTC H-score	71±10	43±21	38±34	59±15	42	0.435
	5T4 Burden/mL	170±77	61±37	50±28	34±12	35±35	0.326
	Cluster weighted 5T4 burden/mL	541±283	184±127	50±28	53±19	61±61	0.163
squamous cell carcinoma	CTC #	55±26	119±114	73±63	NA	1.0	0.992
	CTC H-score	39±15	33±18	37±19	NA	NA	0.971
	Cluster weighted CTC H-score	50±19	57±35	55±11	NA	NA	0.992
	5T4 Burden/mL	161±120	183±161	179±109	NA	4.4	0.992
	Cluster weighted 5T4 burden/mL	297±177	414±307	450±366	NA	4.4	0.671

### Characterization of 5T4- expression in CTCs

#### CTC H-score

The intensity of 5T4 expression in each detected cell was assigned to a category of 0, 1, 2 or 3 by comparing the intensity to a standard curve comprised of characterized control cells with known 5T4 expression (as described in Materials and Methods). A CTC H-score was generated by calculating the percentage of cells per samples that fell into each of these categories (Table 6).

The average CTC H-score in the adenocarcinoma samples was 56±7 per mL of blood. The average CTC H-score in the squamous cell carcinoma samples was 50± 7 per ml of blood. There was no significant difference between the CTC H-score in adenocarcinoma and squamous carcinoma samples (p = 0.705). All data are tabulated (Table 9). There were no significant differences in the H-score of adenocarcinoma and squamous cell carcinoma samples as categorized by grade (Table 10) or clinical stage (Table 11).

#### Cluster-weighted H-score

When assessing the H-score initially, each cluster of cells in a sample was counted as a single entity. A cluster-weighted H-score was also calculated for each sample that contained clusters, as described in Materials and Methods, where the number of cells in each cluster was counted and the 5T4 staining intensity in the cluster was corrected for the number of cells in the cluster. The adenocarcinoma samples had a cluster weighted CTC H-score of 70±8, and the squamous cell carcinoma samples had a cluster-weighted H-score of 65±11 (Table 9). There were no significant differences between these cluster-weighted H-scores (p value = 0.876). There were no significant differences in the cluster-weighted H-score of adenocarcinoma and squamous cell carcinoma samples as categorized by grade (Table 10) or clinical stage (Table 11).

#### 5T4 burden per mL

The total intensity of 5T4 staining in each sample (the sum of 5T4 intensity of each cell in a sample) was calculated to give a value for 5T4 burden/mL (Table 6). The 5T4 burden/mL in the adenocarcinoma samples was 96±33 Fluorescence Units (Table 9). The 5T4 burden/mL in the squamous cell carcinoma samples was 168±57 fluorescence units (Table 4d). There was no significant difference between the mean 5T4 burden/mL in each subtype (p value = 0.255) (Table 9).

A cluster-weighted 5T4 burden was also calculated (Table 6). The cluster-weighted 5T4 burden in the evaluable adenocarcinoma samples was 276+118, compared to 389±131 in the evaluable squamous cell carcinoma samples (Table 9). There was no significant difference between these values (p = 0.255).

There are non-significant reductions in 5T4 burden and cluster-weighted 5T4 burden between poorly differentiated and moderately or well differentiated tumors for both adenocarcinoma and squamous cell carcinoma (Table 10). There were also non-significant reductions in the 5T4 burden and cluster-weighted 5T4 burden in the adenocarcinoma samples (Table 11) as categorized by clinical stage. These parameters did not show a decrease in the squamous cell carcinoma samples (Table 11).

#### Concordance between IHC H-score of tumor and various CTC parameters

Examination of the data set (Table 6) revealed that 33 of the 35 samples (94%) had 5T4 expression in the tumor. Of these 33 samples, 24 (72%) had 5T4 expression in their CTCs. Only 2 samples out of 35 (5.7%) had 5T4 expression in the CTCs with no expression seen in the tumor.

The data were analyzed for correlations (Spearman) between the H-score in the tumor samples and various CTC parameters with regard to the degree of 5T4 expression or the number of CTCs. There was no correlation between the matched blood samples and the tumor when the samples were ranked and correlation calculated in this manner (Table 12).

**Table 12:** Spearman correlation between IHC H-score and various CTC parameters.

Indication	Parameter 1	Parameter 2	Rank R	p-value
NSCLC	IHC score	CTCs per mL Blood	0.025	0.882
	IHC score	CTC H-Score:	-0.150	0.391
	IHC score	Cluster-Weighted H-Score:	-0.142	0.415
	IHC score	Cluster Weighted 5T4 Burden per mL Blood	-0.049	0.785
	IHC score	5T4 Burden per mL Blood	-0.004	0.982
adenocarcinoma	IHC score	CTCs per mL Blood	0.171	0.412
	IHC score	CTC H-Score:	-0.010	0.648
	IHC score	Cluster-Weighted H-Score:	-0.100	0.632
	IHC score	Cluster Weighted 5T4 Burden per mL Blood	0.088	0.674
	IHC score	5T4 Burden per mL Blood	0.083	0.691
SCC	IHC score	CTCs per mL Blood	-0.260	0.470
	IHC score	CTC H-Score:	-0.301	0.399
	IHC score	Cluster-Weighted H-Score:	-0.182	0.614
	IHC score	Cluster Weighted 5T4 Burden per mL Blood	-0.212	0.560
	IHC score	5T4 Burden per mL Blood	-0.091	0.811

**Fig 8:**
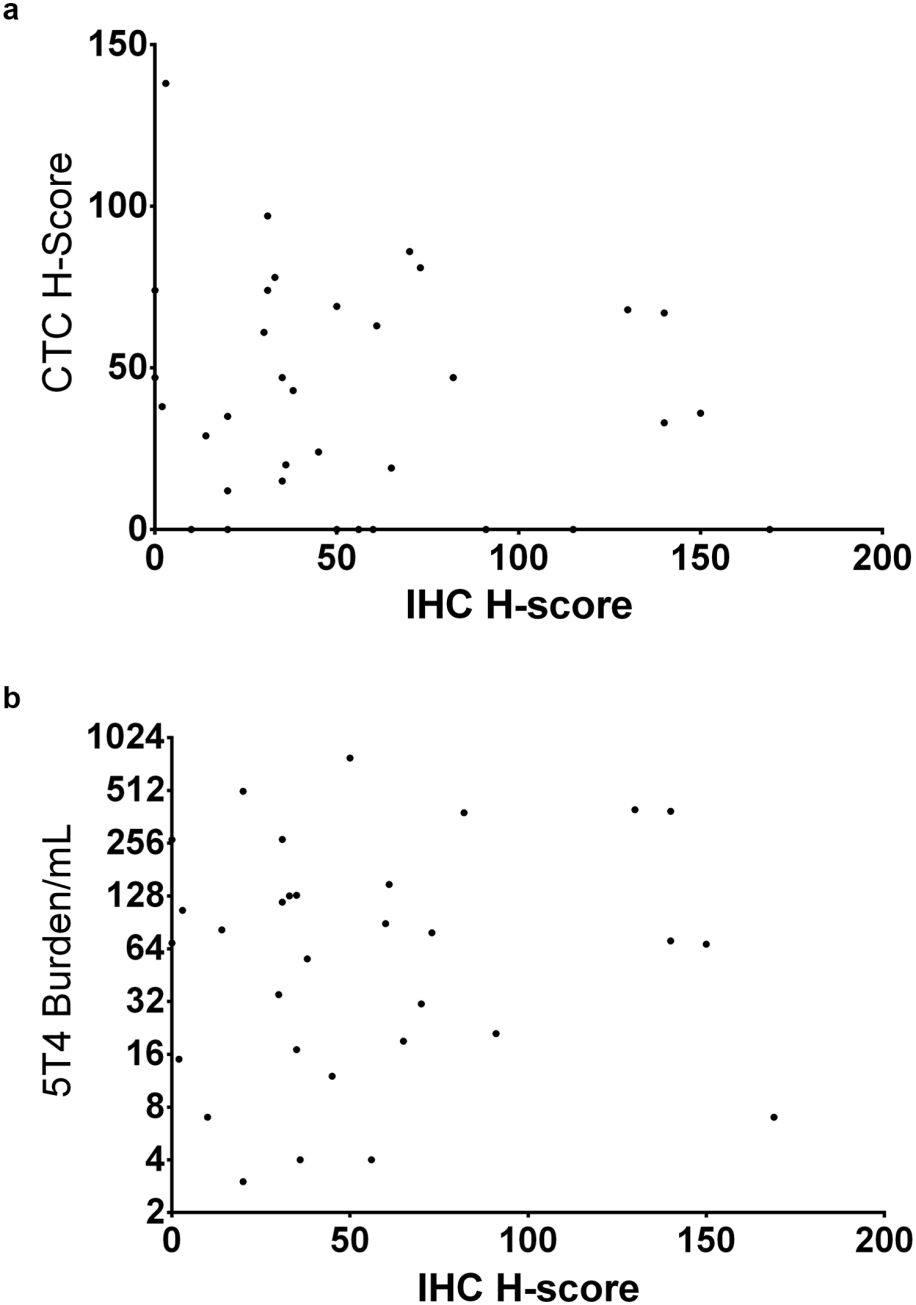
Concordance between IHC H-score and various CTC parameters. **A**- The H score (X-axis) derived for each NSCLC sample, as determined using the 5T4 IHC assay described herein and assessed by a pathologist, was compared to the CTC-H score (Y-axis) as determined by the assay developed to measure 5T4 expression in the CTC compartment. Note the lack of correlation between the degree of expression of 5T4 between the solid tumor and the CTC compartment. **B**- The H score (X-axis) derived for each NSCLC sample, as determined using the 5T4 IHC assay described herein and assessed by a pathologist, was compared to the 5T4 burden/ml in (Y-axis) as determined by the assay developed to measure 5T4 expression in the CTC compartment. Note the lack of correlation between the degree of expression of 5T4 between the solid tumor and the CTC compartment.

Fig 8 shows the lack of correlation between the H score in the tumor and the CTC H score (Fig 8a) and the 5T4 burden/mL (Fig 8b)

## Discussion

Bioinformatics analysis from 1037 samples indicated that 5T4 overexpression is seen in adenocarcinoma and squamous cell carcinoma of lung as compared to non-neoplastic lung. There appeared to be statistically significantly higher expression in squamous cell carcinoma as compared to adenocarcinoma from this *in silico* analysis, although the degree of difference was only 1,6 fold. We have developed an IHC assay that measures 5T4 expression, and this assay was used to profile 5T4 expression in 35 NSCLC samples, representing adenocarcinoma and squamous cell carcinoma subtypes.

The accuracy of an IHC assay is the degree to which the measurement of the visual signal approximates the actual value of what is being measured. The accuracy of the 5T4 IHC assay was established in three parts, using qRT-PCR and western blot as gold standards to first characterize cell lines for 5T4 expression, followed by xenografts, and finally, human tissue samples.

The degree of correlation between qRT-PCR of 5T4 mRNA, quantitative scans of 5T4 western blots, and H-scores of 5T4 IHC was established via Spearman’s rank correlation, which measures the similarity in rank orders of quantitative measurement distributions. Spearman’s rank correlation coefficient (rho) ranges from -1 to +1. Control cell lines expressing a range of 5T4 were identified and characterized by qRT-PCR, western blot, and ICC. The cells maintained their ranking, with regard to 5T4 expression, regardless of the methodology used to rank them.

The ICC and qRT-PCR data generated a rho value of 1.0, the ICC and western blot data generated a rho value of 0.8. These cell lines, when grown as xenografts and stained with an IHC assay, maintained their ranking as relatively negative, low, moderate and high. Xenografts grown from these cell lines were subjected to ischemia and variable times of fixation prior to analysis. Both the mRNA and the protein levels of 5T4, as measured by qRT-PCR and western blot, had only minor fluctuations when exposed to ischemia. Neither ischemia nor variable fixation time impacted the ability of the IHC assay to detect 5T4 in characterized xenografts tissues.

Pulverized tissue extracts and lysates derived from snap frozen human tumors, comprising both stroma and malignant epithelium were evaluated for 5T4 expression using qRT-PCR and western blot. For H-scores, only malignant epithelial 5T4 membrane staining was quantified in formalin-fixed, paraffin embedded fragments of tumors removed from the pestle before pulverization was complete.

Both qRT-PCR and western blots generated digital quantitative values comprising ratios of the 5T4 target to housekeeping genes and/or loading control. IHC analysis generated analog semi-quantitative H-score values comprising intensity and percentage of membrane staining. Although perfect Spearman correlations were not expected, they were anticipated to be greater than zero. H-scores were derived from only the malignant epithelium without regard to the relative proportions of sample epithelium and stroma.

Lung tumor analysis included all samples including subdivisions into squamous cell carcinoma and adenocarcinoma cohorts. Correlation of H-score to western blot data for all lung tumors resulted in a rho values of 0.11, which was lower than anticipated. However, correlation of H-scores to TLDA data resulted in rho values of 0.61. When the analysis was restricted to the squamous cell carcinoma samples, the rho values rose, with a value of 0.66 for IHC to western blot correlation, and 0.62 for IHC to TLDA correlation. Similarly, in the adenocarcinoma sample set, the rho value for IHC to TLDA correlation was 0.69. However, the rho value for IHC to western blot correlation was negative in the adenocarcinoma sample set, suggesting an unknown challenge with analyzing lung adenocarcinoma samples using the current western blot.

In squamous cell carcinoma of lung, these positive rho values were interpreted favorably and served to benchmark the IHC assay accuracy. The negative rho value in lung adenocarcinoma IHC to western blot could not be attributed to a poor quality sample set.

Because lung adenocarcinoma IHC to TLDA correlation was consistent with those seen in lung squamous cell carcinoma, and because there was no visual problem with IHC signal interpretation, we attributed the negative IHC to western blot correlations to a technical problem within the western blot assay itself. We considered the 5T4 IHC assay to be provisionally accurate in lung adenocarcinoma pending additional studies.

A matched set of whole blood, drawn prior to surgery, and prospectively collected NSCLC samples was obtained. The blood was processed as described (see Materials and Methods) for CTC analysis, and the prospectively collected tumor samples were fixed to generate FFPE blocks.

This second set of FFPE NSCLC samples was stained and analyzed using the IHC assay as described. The IHC analysis demonstrated the presence of 5T4 membrane expression in this small cohort of NSCLC tumors and indicated no statistical difference between 5T4 expression in adenocarcinoma and squamous cell carcinoma samples, in disagreement with the *in silico* analysis, which indicated that squamous cell carcinoma samples had a 1.6 fold higher mean expression of 5T4 than adenocarcinoma samples. However, the sample set used for the bioinformatics analysis was much larger than the sample set used for the IHC analysis, which may explain the apparent discrepancy regarding higher expression in squamous cell carcinoma. Furthermore, the average expression of 5T4 in adenocarcinoma and squamous cell carcinoma samples, as measured by IHC, is not significantly altered as a function of clinical stage or grade. Previous assessment of 5T4 expression [2] reported no differences in 5T4 expression between adenocarcinoma and squamous carcinoma [2] and no reported differences in 5T4 expression as a function of tumor stage [2]. Our data presented here are in broad agreement with those previously published data.

We did not see a difference in 5T4 staining between poorly and well differentiated tumors. In the previous study [2] membrane staining of 5T4 was found to be significantly higher in poorly and moderately differentiated tumors versus well-differentiated tumors, with well-differentiated tumors having an average H-score of 31, and poorly differentiated tumors having an average H-score of 64. However those data [2] were generated using a different IHC assay for 5T4, using a different antibody to 5T4, and a much larger sample set, using samples arranged in TMAs, which may account for this differing observation.

An assay has also been developed that enumerates 5T4 expressing CTCs and measures the degree of expression of 5T4 on these CTCs. For the purposes of this study, CTCs are defined as being CD45-/CK+ and having an intact nucleus as defined by DAPI staining. Cytokeratin is an epithelial marker, and it is possible that there is a subset of CTCs that will express reduced levels of cytokeratin, or possibly even mesenchymal specific markers such as vimentin. We have not reported on that population of cells in this current study, and have chosen to focus on CTCs defined as CD45-/CK+/DAPI+. This assay has been used to characterize CTCs in the blood of NSCLC patients who are therapy naïve. Over 90% of these NSCLC samples had detectable CTCs, and in those samples that contained CTCs, on average 50% of those CTCs expressed 5T4. Previous studies using this platform to assess CTCs in blood samples obtained from therapy-naïve NSCLC patients demonstrated that 73% of samples were positive for CTCs (defined as >1 CTC/mL), with a mean of 44.7 CTCs/mL [11]. Those observations are in broad agreement with the data presented here, where we find that the mean number of CTCs in adenocarcinoma samples is 30±98, and in squamous cell carcinoma samples is 55±25.

It has been noted elsewhere that there is no difference between the CTC counts in adenocarcinoma as compared to squamous cell carcinoma [12]. Given that adenocarcinoma and squamous cell carcinoma of the lung both present similar initial patterns of location and metastasize to similar sites, it is unsurprising that there should be no statistical difference in the number of CTCs found in both pathologies. Our data are also in agreement with the observation that there is no significant difference in CTC numbers as a function of tumor stage between patients. We extend those observations to tumor grade. However, in longitudinal studies it has been noted that there is a propensity for increased CTC detection in individuals as disease progresses [13]. We have not conducted a longitudinal study in this report.

Using characterized control cell lines we were able to establish thresholds that defined low, moderate and high expression of 5T4, approximately 50% of detectable CTCs expressed 5T4 to some degree. We were able to assess various metrics including a CTC H-score, and a 5T4 burden. Using these algorithms we show that there are no significant differences between 5T4 expression in squamous and adenocarcinoma samples. Further analysis demonstrated that there were no significant differences in 5T4 expression in the CTCs between various stages and grades of tumor within these indications. It should be noted that expression of 5T4 is strong in all types of NSCLC and in all grades and stages of disease.

We found that 69% of our sample set exhibited 5T4 expression in the tumor and in the CTCs. There were also patients who exhibited 5T4 expression in one compartment but not the other. However, Spearman correlation analysis showed no significant correlation between the degree of expression in the tumor, as measured by H-score, and degree of expression in the CTCs in the matched blood samples. This was true of the entire sample set and the subsets comprised of either the adenocarcinoma or the squamous samples.

There have been several studies investigating the concordance between the phenotype and genotype of tumors as compared to CTCs characterized from the same patient. Some of these studies investigated the concordance between tumor obtained at an earlier time point and CTCs obtained at a later time point. In some of these cases the known phenotypic and genotypic instability of cancer cells [14] may have contributed to the lack of concordance, and in other cases external factors such as therapy may have contributed to the lack of concordance [15].

It is also appreciated that stromal interaction with epithelia cells can modulate the behaviors of carcinomas [16]. The growth and differentiation of an epithelial carcinoma can be influenced by the extracellular matrix, the vasculature, inflammatory cells and the fibroblasts. The importance of these interactions is well established in embryonic development as well as tumorigenesis, and it may be that 5T4 expression is modulated in such a way. This stromal influence may also play a role in the lack of concordance seen between epithelial cells in the tumor and the circulating tumor cell compartment as once an epithelial cell has undergone the epithelial-mesenchymal transition and intravasates, it may escape the direct influence of the stroma, resulting in altered gene expression.

One of the primary goals in assessing target expression in tumors is to select patients who will respond to targeted therapy. The exemplar of such a relationship between quantitative expression of a target in a tumor and response to therapy is Her2 [17]. By using an IHC assay, it has been possible to identify patients who strongly express Her2. Such patients have a better response to anti-Her2 therapy. Further studies confirmed that the greatest clinical benefit occurs in patients who strongly express Her2 [18]. Her2 IHC and FISH are the most widely-used clinically-validated predictive biomarkers for prescribing therapies targeting Her2 [18]. However, even in cases of Her2 overexpression as measured in the tumor, the benefit of anti-Her2 therapy could still be improved upon [18].

Her2 remains the most frequently studied biomarker that has been assessed in both the tumor and in the CTC compartment [19]. It is observed that there is discordance observed between Her2 expression in the tumor and in CTCs [19, 20].

It should be noted that in a recent study, patients with Her2 positivity in the tumor and in the CTC compartment demonstrated an improvement in PFS as compared to patients with Her2-positive tumors but no Her2 detected in the CTC compartment, indicating that information obtained from CTC analysis can help predict response [21].

There is a clinical trial currently underway (DETECT III, NCT01619111) to evaluate whether patients with Her2 positivity in the CTC compartment but undetectable Her2 in the tumor compartment may benefit from anti-Her2 therapy. It will of interest to compare the response rates in the clinical trial with the degree of 5T4 expression in the tumor as well as the degree and extent of 5T4 expression levels in the CTCs, as a potential correlation may suggest that 5T4 positive CTCs may be key contributors to tumor metastases and malignancy.

In conclusion, we have developed assays to measure the degree of 5T4 in both tumor tissue and in CTCs. We demonstrated that 5T4 is measurable in both compartments in patients with NSCLC. We showed no statistical differences in the degree of 5T4 expression with regard to subtype, grade or stage of disease either in the tumor or in the CTC compartment. We also demonstrated that a majority of patients with measurable 5T4 in tumor also have detectable 5T4 in CTCs. These assays, suitably validated for clinical use, will allow us to assess the value of 5T4 expression (in either tumor or CTCs) and determine if there is a threshold value that can serve as a predictive biomarker with regard to response to treatment options that target 5T4.

## Materials and methods

### Bioinformatics analysis of 5T4 expression in human tumors and cell lines

To assess the 5T4 mRNA expression level in subtype of NSCLC (squamous cell carcinoma and adenocarcinoma), Level 3 RNASeq expression data (TPM, transcript per million) were accessed from TCGA data portal (The Cancer Genome Atlas, https://tcga-data.nci.nih.gov/docs/publications/tcga/) (tumors and normal). Differential expression of 5T4 expression level in Lung adenocarcinoma (n = 526) and Lung squamous cell carcinoma tumor types (n = 501) were compared to their matched normal samples (n = 58 and 51 respectively). The messenger ribonucleic acid (mRNA) expression level of 5T4 in cell lines from the CCLE (reference) was ranked based on the log2 signal values on the Affymetrix Human Genome U133 Plus 2.0 chip from the Cancer Cell Line Encyclopedia (CCLE) database. Cell line 5T4 mRNA expression levels were ranked lowest to highest, the cohort was divided into quartiles, and representative cell lines from each quartile were selected as relatively negative, low, moderate, and high control cell lines for diagnostic assay development, based in part on their ability to grow as xenografts.

### Representative cell lines

All cell lines were purchased from American Type Culture Collection (ATCC) (Manassas, VA). The following cell lines were used in this study: NCI-H460 (ATCC HTB-177) Lot No. 59378191, NCI-H2122 (ATCC CRL-5985) Lot No. 60434345, NCI-H1975(ATCC CRL-5908) Lot No. 59300809, and NCI-H226 (ATCC CRL-5826) Lot No. 59900056. All cell lines were tested and authenticated by IDEXX RADIL Laboratories (Columbia MO). Services/tests performed: Cell Check 16; IMPACT I PCR Profile; Corynebacterium bovis-PCR. PCR evaluation for: Corynebacterium bovis, Ectromelia, EDIM, Hantaan, K virus, LCMV, LDEV, MAD1, MAD2, mCMV, MHV, MNV, MPV, MTV, MVM, Mycoplasma pulmonis, Mycoplasma sp., Polyoma, PVM, REO3, Sendai, TMEV GDVII, and Species-specific PCR evaluation. The samples were confirmed to be of human origin and no mammalian interspecies contamination was detected. The alleles for 16 different markers were determined, and the results were compared to the alleles reported for each cell line. The base medium for the 5T4 NSCLC cell lines was Life-Technologies formulated RPMI-1640 Medium, Catalog No. 11875–085. The following components were added to the base medium: fetal bovine serum to a final concentration of 10% (v/v), Sigma Aldrich, Catalog No. F2442. Cultures were maintained in a humid incubator at 5% CO2 and 37°C.

### Xenograft tumor growth for PAV

All experimental animal procedures complied with the Guide for the Care and Use of Laboratory Animals (Institute for Laboratory Animal Research, 1996) and were approved by the Pfizer Global Research and Development Institutional Animal Care and Use Committee (IACUC). Mice were obtained from Charles River Breeding Laboratories (Wilmington, MA) and acclimatized for a minimum 72-hour period prior to use. Female Nude Crl:NU(NCr)-Foxn1nu mice were used as the animal model. Tumor cells (2x106) growing in the mid-logarithmic phase were suspended in 100 μl PBS with 50% (v/v) Culture Basement Membrane Extract, Path Clear, Trevigen Catalog No. 3432-005-01 and implanted sc in the right flank of mice. Tumor sizes in these models were assessed by caliper measurements using the formula V = (W^2 × L)/2 where V is tumor volume, W is tumor width, L is tumor length. Tumors were harvested when tumor volumes reached an average of 500–650 mm3. Time to size was model dependent and ranged from 15–60 days post implant.

Xenograft tumors were collected in triplicate and each one bisected along the midline, placed into individual tissue embedding cassettes and subjected to either 0 hours ischemia or 6 hours ischemia (by submersion in room temperature saline). Samples were then fixed for either 8 hours or 24 hours, resulting in tissues that had been subjected to 4 conditions; 0 hours ischemia/8 hours fixation, 0 hours ischemia/24 hours fixation, 6 hours ischemia/8 hours fixation, and 6 hours ischemia/24 hours fixation. Samples to be analyzed by in situ hybridization (ISH) or IHC were subjected to these conditions in order to mimic ischemia and fixation times routinely encountered in post-operative collections. Samples were processed according to the protocol outlined in Table 2. Post-ischemia/pre-fixation samples collected for downstream analysis including qRT-PCR and western blot were collected in triplicate and bisected into equal halves along the midline and treated to either 0 hours or 6 hours of ischemia before being snap frozen and processed as outlined above.

Snap frozen tumor (500mg) was kept on dry ice until processed. Tumor tissue was placed in the liquid nitrogen filled base of a chilled Cell Crusher. Once the liquid nitrogen had nearly evaporated, the top of the chilled Cell Crusher was placed on top of the frozen tissue and the apparatus struck with a hammer until the tissue was pulverized. Pulverized tissue was utilized for protein lysate or ribonucleic acid (RNA) processing.

### Measurement of gene expression by qRT-PCR

Representative cell lines were grown (in recommended growth media) to 70–80% confluence. Cells were lysed with 2mL of RLT buffer (Qiagen, Catalog No. 79216). RNA isolation was performed using the Qiagen RNeasy Plus Mini Kit (Catalog No. 74134) according to manufacturer’s instructions. RNA was isolated from powdered xenograft PAV material using Qiagen RNeasy Plus Mini Kit (Catalog No. 74134), according to manufacturer’s instructions. RNA quantitation for representative cell lines and xenograft tumors was performed using the Thermo Scientific Nanodrop-8000 (Thermo-Fisher Scientific, Waltham, MA), according to standard protocol. The remaining sample was stored at -80°C until needed. Isolated RNA samples were reverse transcribed to complementary deoxyribonucleic acid (cDNA) using the Life Technologies, High Capacity RNA-to-cDNA Kit (Life Technologies, Catalog No. 4387406) following the standard protocol outlined in the manufacturer’s directions. The cDNA plates were stored at -20°C until qRT-PCR analysis. The qRT-PCR reaction was performed using the TaqMan Probe-Based Gene Expression Analysis and ABI ViiA7 Real-Time PCR (RT-PCR) Systems (Life Technologies, Carlsbad CA). Samples and were run in triplicate for each probe set. The qRT-PCR master mix was prepared for each sample according to manufacturer’s instructions (Life Technologies, Catalog No. 4352042). The plate was sealed and centrifuged briefly. Care was taken to ensure that the plate was at room temperature for 10 minutes prior to the run. Default thermal cycling conditions were as follows: The qRT-PCR reaction was run on an ABI ViiA7 thermal cycler in three stages; 2 minutes at 50°C, 10 minutes at 90°C and 40 cycles of 15 seconds at 90°C followed by 1 minute at 60°C. Quantitation was assessed using the comparative cycle threshold (Ct) method, (2^-[delta][delta]Ct method, where [delta][delta]Ct = [delta]Ct,sample—[delta]Ct,reference) relative to a Universal RNA Control. The Ct values of both the calibrator and the samples of interest were normalized to the listed set of endogenous housekeeping genes.

### Western blot analysis of representative cell lines and xenograft tumors

Cells were grown to 70–80% confluence and rinsed with ice cold phosphate buffered saline (PBS) (Life Technologies Catalog No. 10010–023). Rinsed cells were lysed in RIPA buffer (Thermo Fisher, Catalog No. 89901) supplemented with proteinase inhibitors (Roche Applied Science Catalog No. 11-836-153-001) and phosphatase inhibitors (Roche Applied Science, Catalog No. 04-906-845-001) according to manufacturer’s instructions. The cellular homogenate was clarified by centrifugation and the resultant supernatant was stored and analyzed. Snap frozen tumor tissues for protein lysate were further pulverized to powder in a liquid nitrogen cooled Cell Crusher. Samples (100mg) were transferred into pre-cooled collection tubes (FastPrep Metal Bead Lysing Matrix (MP Biomedicals, Catalog No. 6925–050) containing RIPA lysis buffer. Samples were pulsed twice according to manufacturer’s directions. Resultant homogenate was clarified by centrifugation, and the supernatant was decanted away from precipitate. Protein content was quantified using a colorimetric BCA assay (Thermo Fisher Scientific, Catalog No.PI-23-221). The resultant supernatant was stored and analysed. Samples were reduced (XT Reducing agent (20X), Bio-Rad, Catalog No. 161–0792) and electrophoresed using a 4–12% Bis-Tris gel (Bio-Rad Catalog No. 345–0124) using MOPS running buffer (Bio-Rad, Catalog No. 161–0788) at 150V for 1.5 hours. Resolved protein was then transferred to 0.2 μm nitrocellulose membranes (Bio-Rad Catalog No. 162–0233) using NuPage transfer buffer (Life Technologies Catalog No. NP-0006-1) at 100V for 1.5 hours. The western blot membrane was removed from the transfer apparatus, blocked using a 5%(w/v) non-fat dry milk solution (Cell Signaling, Catalog No. 9999) in TBST solution (Cell Signaling, Catalog No. 9997) at room temperature for 1 hour and incubated with the primary antibody (EPR 5529, Abcam, Catalog No. ab134162) at a dilution of 1:1000 in blocking solution overnight at 4°C. The blot was then rinsed in TBST solution and incubated with a secondary antibody (anti-rabbit IgG (H+L), Thermo Fisher, Catalog No. 32460) diluted 1:3000 in blocking solution for 1 hour at room temperature. The blot was rinsed in TBST solution and developed using SuperSignal West Dura (Thermo Fisher Scientific, Catalog No. 37071) for 5 minutes, protected from light. The image was acquired using a Bio-Rad Chemi-Doc XRS (Bio-Rad, Hercules, CA) chemiluminescent Imaging Detection system and acquired images analyzed using Image Lab software Version 5.1 (Bio-Rad, Hercules, CA). Membranes were then stripped for 10 minutes using Restore Plus Western Blot Stripping Buffer (Thermo Fisher, Catalog No. 46430), rinsed in PBS, blocked for 1 hour in blocking solution at room temperature, and re-probed for GAPDH using clone 14C10 antibody (Cell Signaling, Catalog No. 2118). The reprobed blot was developed as described above.

Cells were grown to 80–90% confluence in appropriate media in Corning T-225 flasks (Corning, Catalog No. 431082). Cells were resuspnded in 500 mL of media and decanted into a Cell-Bind Hyper-Flask (Corning, Catalog No. 10020). Once cells were again at 80–90% confluence (4–10 days) they were harvested with 50 ml TrypLE Express dissociation reagent (Life Technologies, Catalog No.12605-10), centrifuged, rinsed, and centrifuged again to pellet the cells. The cell pellet was carefully removed from the centrifugation tube using a spatula onto a piece of lens paper and the paper folded and placed into 10% neutral-buffered formalin (NBF) for 18 hours at room temperature. The cell pellet was the processed on a Leica ASP 300s (Leica, Buffalo Grove, IL) tissue processor according to the protocol outlined in (Table 1).

Xenograft tumors were collected in triplicate and each one bisected along the midline, placed into individual tissue embedding cassettes, and subjected to either 0 hours ischemia or 6 hours ischemia (by submersion in room temperature saline). Samples were then fixed for either 8 hours or 24 hours, resulting in tissues that had been subjected to 4 conditions; 0 hours ischemia/8 hours fixation, 0 hours ischemia/24 hours fixation, 6 hours ischemia/8 hours fixation, and 6 hours ischemia/24 hours fixation.

### Prospectively collected NSCLC samples

An analytical validation sample set of prospectively collected NSCLC human tumors was obtained from a variety of sources, including the Pfizer tumor bank and commercial vendors. All commercially sourced human biospecimens, including blood samples and matched tumor tissues, were obtained from Conversant Bio. Biospecimens distributed by Conversant Bio are collected, processed, and distributed in full ethical and regulatory compliance with the Sites from which human biospecimens are collected. This includes independent ethical review, Institutional Review Board approval (where appropriate), independent regulatory review, and Conversant Bio ethical review for all of Conversant Bio’s collection Sites. All tissues were obtained within applicable laws.

All other human biospecimens in this study were obtained from Pfizer's internal Tissue Bank which obtained anonymized specimens from commercial and academic sources that collected the specimens with donor consent under Institutional Review Board-approved procedures. These tumors were snap-frozen at the time of collection.

Frozen Tissue (~ 500 mg) was kept on dry ice until processed. Tissue was placed in the liquid nitrogen filled base of a chilled Cellcrusher (CellCrusher Limited), and once the liquid nitrogen had nearly evaporated, the top of the chilled Cellcrusher was placed on top of the frozen tissue, and the apparatus struck with a hammer until the tissue was fragmented. Random pieces of crushed tissue were chosen for fixation, protein lysate processing or RNA processing. Frozen fragmented human tumor tissue was transferred to 10% NBF, at room temperature, and allowed to fix for 24 hours with gentle agitation on a bench-top rocker. Tissues were then processed on a Tissue-Tek VIP tissue processor (Sakura Finetek USA). This sample set was used to analytically validate the IHC assay.

A second set of NSCLC tissue and matched blood samples was obtained prospectively (ConversantBio, Huntsville, AL), from therapy naïve patients at initial diagnosis and processed to generate FFPE blocks at the site and time of collection. All sample collection was appropriately consented. A matched blood sample was obtained from each patient immediately prior to biopsy. Each sample was accompanied by a pathology report giving details of indication, stage and grade. This sample set was used to determine the correlation of the IHC assay to the CTC assay with regard to presence of and degree of 5T4 expression.

### IHC assay

Tissue sections (4μm) were obtained from each block. Paraffin was removed and samples were re-hydrated using standard xylene/ethanol immersion. Antigen retrieval was achieved using Leica ER2 solution (Leica BioSystems, Buffalo Grove, IL) at 97°C for ten minutes. Slides were then blocked using the Leica Bond refine detection kit (cat # DS9800) followed by Cyto Q background buster (Innovex Biosciences, Richmond, CA) on a Leica Bond III (Leica Microsystems, Buffalo grove, IL). Slides were then incubated for 20 minutes in a rabbit monoclonal antibody(36ng/mL) raised against a peptide region within 5T4 (AbCam. Burlingame, CA cat # ab134162). Antibody was detected and slides were counterstained using the Leica Bond refine detection kit (DS9800). Stained sections were assessed and scored by an anatomic pathologist to generate H-scores.

### CTC assay protocol

Blood was collected into Cell Free DNA BCT tubes (Streck) by standard venipuncture and shipped at ambient temperature to Epic Sciences (San Diego, CA). Red blood cells were lysed within 48 hours of each blood draw, and remaining nucleated cells were deposited onto proprietary glass slides at a target density of 3×106 cells/slide. Slides were then frozen at -80°C for long term preservation. CTC slides stored using this approach are stable for over 1 year (unpublished data).

CTC analysis was performed in batch. Two slides from each patient sample were thawed and stained with an immunofluorescent assay to distinguish CTCs from WBCs. Samples were additionally treated with anti-5T4 antibody (R&D Systems cat no. AF4975) to characterize CTC candidates by 5T4 expression. Epic’s proprietary CTC detection systems were utilized to scan stained slides in 4 fluorescent channels and analyze the resulting imagery to identify CTC candidates. All candidates were then reviewed by trained technicians, and CK+/CD45- cells with intact DAPI+ nuclei exhibiting tumor-associated morphologies were classified as CTCs. Quantitative measurements of 5T4 expression were obtained by Epic’s automated image analysis system and provided for each CTC identified.

### Control materials and establishment of NLMH thresholds for the CTC assay

Human lung cancer cell lines were screened for 5T4 expression by IHC staining and 4 cell lines were identified with various expression levels: H460 (negative-low), H2122 (low), H1975 (medium), H226 (high). All cell lines were cultured at 37°C in 5% CO2 using RPMI 1640 media (Gibco cat no. A10491) supplemented with 10% fetal bovine serum (Gibco cat no. 10082–147) and 5% penicillin/streptomycin (Gibco cat no. 15070–063). Live cultured cells were then spiked into healthy donor blood samples at a density of 1 tumor cell per 10,000 WBCs. The spiked samples were prepared and analyzed at Epic Sciences using the aforementioned methods. Enough spiked slides were created to provide controls for each batch of patient slides stained in this study. Individual cells’ 5T4 expression was plotted, and suspected 5T4 expression trends were confirmed. NLMH thresholds specific to Epic’s platform were created to separate cell line populations into expression groups that were consistent with the previous IHC analysis. These thresholds were as follows: negative = 0.00–2.99, low = 3.00–7.99, medium = 8.00–19.99, high ≥ 20.00. Using these thresholds, H-scores could be calculated for spiked cell populations on the Epic platform. Within a range of 0 (negative) to 300 (highly positive), the control cell lines produced the following H-scores: H460 = 58, H2122 = 102, H1975 = 201, H226 = 256.

### Statistical methods

All statistical analysis was performed using either SpotFire 3.3.3 (build 3.3.3.14) (TIBOC, Boston, MA) or GraphPad Prism version 6.03 for Windows (GraphPad Software, San Diego, CA).
